# Awareness of gestational diabetes mellitus foetal-maternal risks: an Italian cohort study on pregnant women

**DOI:** 10.1186/s12884-021-04172-y

**Published:** 2021-10-09

**Authors:** Paola Quaresima, Federica Visconti, Fabiana Interlandi, Luigi Puccio, Patrizia Caroleo, Giuseppina Amendola, Michele Morelli, Roberta Venturella, Costantino Di Carlo

**Affiliations:** 1 Unit of Obstetrics and Gynaecology, Department of Clinical and experimental Medicine, “Magna Græcia” University of Catanzaro, Viale Europa, 88100 Catanzaro, Italy; 2 Complex Operative Structure Endocrinology-Diabetology, Pugliese-Ciaccio Hospital, 8100 Catanzaro, Italy; 3 Complex Operative Structure Obstetrics and Gynaecology, Annunziata Hospital, 87100 Cosenza, Italy

**Keywords:** Gestational diabetes, Awareness, Intrauterine foetal death

## Abstract

**Background:**

Gestational diabetes mellitus (GDM) incidence is increasing worldwide. It represents a major risk factor for adverse foetal-maternal outcomes. Awareness among women in regard to GDM-related risks (in particular foetus ones) has been proven to have an impact on compliance with recommendations. Therefore we aimed to evaluate the efficacy of our post-diagnosis counselling, that informs affected women of the GDM related risks for complications, in determining an adequate level of understanding.

**Method:**

This is a cohort study involving 400 women undergoing the 24-28 weeks 75 g oral glucose tolerance test. Two hundred women diagnosed with GDM received the post-diagnosis counselling (treatment group) and two hundred women diagnosed without did not receive any counselling (control group). Both populations were surveyed with a 5 question questionnaire regarding their awareness about GDM foetal-maternal related risks. Their level of education about GDM foetal-maternal related risks, estimated according to the number of correct answers, was scored as: primary (score 0-1), secondary (score 2-3) or tertiary (score 4-5).

**Results:**

Most of the women in the treatment group after receiving the post-diagnosis counselling have demonstrated a secondary level of education 132/200 (66%). Their mean level of awareness was higher in comparison to the control group 2.6 ± 1.8 (SD) versus 2.14 ± 1.8 (SD) *p value = 0.012*. In particular, they’ve demonstrated to be more aware of the risks for the foetus to become macrosomic (*p = 0.004*) or to die in utero (*p = 0.0001*). A high level of education and to have had previous pregnancies positively affected correct answers.

**Conclusions:**

Our post-diagnosis counselling has played a role in improving women awareness about GDM foetal-maternal related risks. Future study will explore the impact of women’s level of awareness on glycaemic control.

## Background

Gestational diabetes mellitus (GDM) is defined as any degree of glucose intolerance with onset or first recognition during gestation. It is the most common medical disorder diagnosed during pregnancy [1, 2]. In 2019, the International Diabetes Foundation estimated that 16% of women giving births had some form of hyperglycaemia in pregnancy, with an estimated 85.1% due to GDM. Moreover, GDM incidence is increasing worldwide because of progressing trends in obesity and advancement of maternal age among women during childbearing age [3].

In Italy, GDM incidence ranges from 10 to 14% in the Northern regions to up to 28% in the Southern ones [4, 5]. Current national guidelines recommend a 75 g Oral Glucose Tolerance Test (OGTT) at 24-28 weeks of gestation for women considered to be at risk for GDM, preceded by an early testing at 16-18 weeks of gestation for women considered to be at “high” risk [6–8]. If the early test results are negative, a second one is recommended at 24-28 weeks of gestation. GDM is a topic of great interest because it represents a major risk factor for adverse foetal-maternal outcomes such as preeclampsia, preterm birth, foetal macrosomia, polyhydramnios, shoulder dystocia, Caesarean section, neonatal respiratory distress, neonatal hypoglycaemia and perinatal mortality. An appropriate management of this disorder (adequate counselling, self-glucose monitoring, diet, physical activity, and eventually medication) is crucial for a favourable pregnancy outcome [1, 8].

Untreated GDM carries significantly higher risks for all of the complications mentioned previously [9]. Among the factors that have an impact on an appropriate management of this disorder, we have temporal, physical, social constraints, and, a pivotal role is played by maternal awareness regarding GDM related risks in particular foetal ones. It has been proven that a low level of awareness is correlated with worse glycaemic control which, in turn, is associated with a higher incidence of adverse pregnancy outcomes [10]. Limited data is available regarding women awareness of GDM [10–14]. Considering the high prevalence of this disorder in our population, it emerges as a crucial endeavour to realize an effective post-diagnosis counselling with the aim to spread female awareness of the foetal-maternal risks related to it. Therefore, in the present study, we evaluated the efficacy of our post-diagnosis counselling in determining an adequate level of understanding about the foetal-maternal related risks. With this purpose, we compared the level of awareness about GDM related risks between women diagnosed with, that had received the specific post-diagnosis counselling (considered as treatment group) and women diagnosed without who did not receive any counselling (considered as a control group).

## Materials and methods

To evaluate the efficacy of our post-diagnosis counselling for GDM, we realized a cohort study in the setting of a referral centre for diabetes in Catanzaro at the Pugliese Ciaccio Hospital (Calabria, Italy), from July 2020 to March 2021. All consecutive pregnant women attending the Unit to undergo the oral glucose tolerance test for GDM at 24-28 weeks of gestation were invited to participate in the study, and those who accepted to be enrolled where considered eligible.

Considering that in a nine months’ period of time we observe approximately 200 positive oral glucose tolerance test result for GDM we decided to stop recruitment when the first consecutive 200 women diagnosed with GDM and the first consecutive 200 women with a negative test result had been considered eligible. An informed consent was acquired. The study was approved by the local ethics committee. Among the eligible women, those diagnosed with GDM received a structured counselling about incidence, pathophysiology, risk factors, foetal-maternal risks, glucose monitoring, and pregnancy management. In more detail, the described foetal-maternal risks related to GDM have been: foetal macrosomia (a new-born much larger than average); Shoulder dystocia (the difficulty to deliver baby’s shoulder after its head has emerged); Polyhydramnios (the pathological increase in the amniotic fluid volume); Preeclampsia (an hypertensive disorder of pregnancy associated with signs of damage to other organ systems), and the intrauterine foetal death (the diagnosis of foetal death in utero) [8]. The counselling was performed in every case by the same team, which was composed by a diabetologist and an obstetrician with a special interest in GDM diagnosis and management (P.C. and P.Q.). Each one-time session occurred in a quiet room and lasted 15-20 min for each patient. Both groups of women, those diagnosed with GDM receiving the post-diagnosis counselling (treatment group) and those diagnosed without who had not received any counselling (control group) answered to a questionnaire with five multiple-choice answers regarding their awareness on GDM related foetal-maternal risks. The questionnaire was designed by the authors, diabetologists and obstetricians, with a special interest in GDM diagnosis and management: L.P.; P.C.; P.Q.; F.V.) (Table 1).

**Table 1:** Questionnaire questions

N°	Questions:	Yes	No
1	Does GDM increases the risk for the foetus to become macrosomic?		
2	Does GDM increases the risk for the foetus to experience a shoulder dystocia?		
3	Does GDM increases the risk for polyhydramnios?		
4	Does GDM increases the risk for the maternal risk for Preeclampsia?		
5	Does GDM increases the risk for intrauterine foetal death?		

Each affirmative response (Yes) was given a score of 1 whereas each negative response (No) was given a score of 0. The level of education ranged from zero to five according to the number of correct answers. A score of 0-1 was considered as primary level of education; A score of 2-3 was considered as secondary level of education; A score of 4-5 was considered as tertiary level of education; Additional information was collected: age, body mass index (BMI) at the first trimester of pregnancy, parity, nationality (Italian vs. other European nationalities vs. extra-European), level of education (low level of education: illiterate or compulsory school versus high level of education: high school or college), family history of T2DM, previous pregnancies complicated by GDM and /or foetal macrosomia. After completing the questionnaire, all pregnant women had the chance to pose their questions about GDM to the counselling team.

### Statistical analysis

Continuous variables are expressed as median and interquartile range (IQR), and categorical variables as numbers and percentages. After testing for normality of all continuous variables by the Shapiro-Wilk normality test, the non-parametric Mann-Whitney test was used for comparisons of continuous variables. The 2-tailed Fisher exact test was used for comparisons of proportions. Statistical significance was fixed at an alpha level of 0.05. A binomial logistic regression analysis was used to evaluate the effect of confounders (maternal age, BMI, level of education, previous pregnancies, family history for diabetes, previous GDM and or macrosomic foetus) as possible predictors of an affirmative answer for each question, providing Odds Ratios (ORs) with 95% confidence bounds. Statistical analyses were performed with SPSS 20.0 software (SPSS Inc., Chicago, IL, USA).

## Results

During the study period, we performed 800 oral glucose tolerance test, the first consecutive 200 women diagnosed with GDM and the first consecutive 200 women diagnosed without GDM who accepted to participate in the study were included. During the recruitment time, 15 women diagnosed with GDM and 5 without refused to participate. The sample size has been of 400 women.

All women who accepted to participate completed entirely the questionnaire, no missing data. Women characteristics are illustrated in Table 2.

**Table 2:** Women characteristics

	Treatment group: women diagnosed with GDM (n = 200)	Control group: women without GDM (***n*** = 200)	P value
**Maternal Age**	32 (28-36)	29.5 (27-34)	**0.001**
**Low Level of education:** illiterate or compulsory school	29 (14.5%),	36 (18%)	0.3
**High Level of education:** high school or college	171 (85.5%)	164 (82%)	0.34
**At least one previous pregnancy**	100 (50%)	98 (49%)	0.919
**Previous GDM**	34 (17%)	4 (2%)	**< 0.001**
**Previous macrosomic foetus**	15 (7.5%)	5 (2.5%)	**0.038**
**Family history for Diabetes**	69 (34.5%)	16 (8%)	**< 0.001**
**BMI** (kg/m^2)	29.5 (26 – 33)	27 (24 – 30)	**0.001**

*GDM* Gestational Diabetes Mellitus, *BMI* Body mass index

The two populations were significantly different in terms of age, BMI, family history for diabetes, obstetric history of GDM or macrosomic foetus. As predicted, affected women were older, heavier, with family history for Diabetes and previous obstetric history positive for GDM and/or macrosomic foetuses. The level of awareness of the treatment group resulted to be: a primary level of education (score 0-1) for 65/200 (32.5% of women), a secondary level of education (score 2-3) for 67/200 (33.5% of women), and a tertiary level of education (score 4-5) for 68/200 (34% of women). At least two affirmative answers were given by 132/200 (66% of women).

**Fig. 1:**
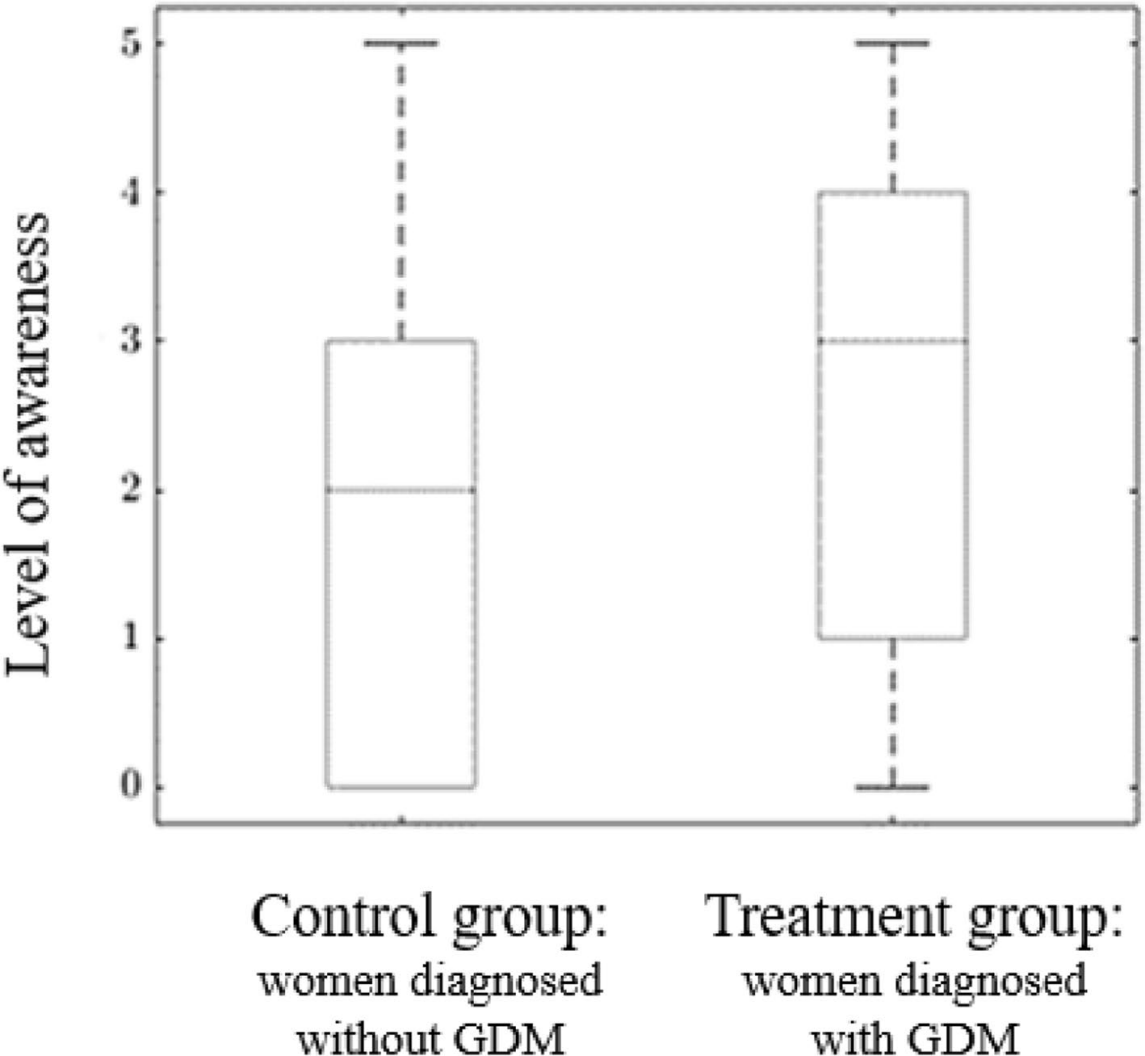
Mean level of awareness according to the number of correct answers (0-5) between the groups of women diagnosed with (treatment group) or without GDM (control group)

The mean level of awareness (0-5) was significantly higher for the treatment group in comparison to the control 2.6 ± 1.8 (SD) versus 2.14 ± 1.8 (SD); *p* value = 0.012 (Fig. 1).

As detailed in Table 3, a significant difference in terms of the number of correct answers was found for both question 1 and question 5, with a higher percentage of aware women in the treatment group. Question 1 (Does GDM increases the risk for the foetus to be macrosomic?) p value = 0.004 and Question 5 (Does GDM increases the risk for intrauterine foetal death?) p value = 0.0001.

**Table 3:** Questionnaire questions answers

	Treatment group: women diagnosed with GDM (n = 200)	Control group: women without GDM (n = 200)	P value
Does GDM increases the risk for the foetus to become macrosomic?	135/200 (67.5%)	105/200 (52.5%)	**0.004**
Does GDM increases the risk for the foetus to experience a shoulder dystocia?	58/200 (29%)	49/200 (24.5%)	0.36
Does GDM increases the risk for polyhydramnios?	86/200 (43%)	75/200 (37.5%)	0.3
Does GDM increases the risk for the maternal risk for Preeclampsia?	139/200 (69.5%)	139/200 (69.5%)	1
Does GDM increases the risk for intrauterine foetal death?	100/200 (50%)	61/200(30.5%)	**0.0001**

*GDM* Gestational Diabetes Mellitus, *BMI* Body mass index

Binomial logistic regression analysis was used to evaluate individual effects of potential predictors or confounders, on an affirmative answer for each question.

Question 1 “Does GDM increases the risk for the foetus to become macrosomic?”: A correct answer was more likely in the presence of: a high level of education OR 2.09, CI 1.52-3.16, *p* < 0.001 and the fact of having had previous pregnancies OR 2.4 and CI 1.49-3.93, *p* = 0.001;

Question 2 “Does GDM increases the risk for the foetus to experience a shoulder dystocia?”: A correct answer was more likely in the presence of: a high level of education OR 2.1 and CI 1.31-2.89, *p* < 0.001 and the fact of having had previous pregnancies OR 1.92 and CI 1.0-2.83, *p* = 0.04; Question 3 “Does GDM increases the risk for polyhydramnios?”: A correct answer was more likely in the presence of: a high level of education OR 2.1 and CI 1.46-2.98, *p* < 0.001 and the fact of having had previous pregnancies OR 1.84 and CI 1.21-3.09, *p* = 0.01.

Question 4 “Does GDM increases the risk for the maternal risk for Preeclampsia?”: A correct answer was more likely in the presence of: a high level of education OR 2.43 and CI 1.65-3.63 p < 0.001 and the fact of having had previous pregnancies OR 2.67 and CI 1.55-4.38 *p* < 0.001.

Question 5 “Does GDM increases the risk for intrauterine foetal death?”: A correct answer was more likely in the presence of: a high level of education OR 2.2, CI 1.71-3.63, p < 0.001 and the fact of having had previous pregnancies OR 1.74 and CI 1.12-2.95, *p* = 0.028;

A description of Odds Ratios, Confidence Intervals and *p* value is available on Table 4.

**Table 4:** Binomial logistic regression analysis

	Does GDM increases the risk for the foetus to become macrosomic?		Does GDM increases the risk for the foetus to experience a shoulder dystocia?		Does GDM increases the risk for polyhydramnios?		Does GDM increases the risk for the maternal risk for Preeclampsia?		Does GDM increases the risk for intrauterine foetal death?	
Variables	OR (CI 95%)	p	OR (CI 95%)	p	OR (CI 95%)	p	OR (CI 95%)	p	OR (CI 95%)	p
Maternal age	0,97 (0.93-1.02)	0,369	0,97 (0.92-1.02)	0,372	0,97 (0.92-1.01)	0,212	0,97 (0.94-1.03)	0,426	0,97 (0.89-0.98)	0,372
BMI	0,81 (0.72-1.02)	0,642	0,99 (0.97-1.09)	0,989	0,48 (0.45-1.09)	0,077	0,81 (0.77-1.09)	0,645	0,48 (0.38-0.88)	0,086
Level of education	2,09 (1.52-3.16)	**< 0,001**	2,10 (1.31-2.89)	**< 0,001**	2,10 (1.46-2.98)	**< 0.001**	2,43 (1.65-3.63)	**< 0,001**	2,20 (1.71-3.63)	**< 0,001**
Previous pregnancies	2,40 (1.49-3.93)	**0,001**	1,92 (1.00-2.83)	**0,045**	1,84 (1.21-3.09)	**0,011**	2,67 (1.55-4.38)	**< 0.001**	1,74 (1.12-2.95)	**0,028**
Previous GDM	1,13 (0.49-3.13)	0,786	1,61 (0.71-3.85)	0,280	1,32 (0.59-3.06)	0,513	2,80 (0.81-6.87)	0,090	0,49 (0.36-0.88)	0,086
Previous macrosomic foetus	0,93 (0.28-2.42)	0,902	1,61 (0.59-4.67)	0,374	0,80 (0.26-2.09)	0,685	0,54 (0.16-1.57)	0,325	1,60 (0.48-3.95)	0,392
Family history for T2DM	1,09 (0.58-1.84)	0,753	0,62 (0.36-1.32)	0,167	1,13 (0.66-2.03)	0,659	1,09 (0.56-1.86)	0,780	1,05 (0.59-1.89)	0,859

*GDM* Gestational Diabetes Mellitus, *BMI* Body mass index, *T2DM* type 2 diabetes mellitus

A summary of results is available on Table 5.

**Table 5:** Results

Significant difference in mean level of awareness between women diagnosed with GDM (treatment group): 2.6 ± 1.8 (SD) and women diagnosed without GDM (control group): 2.14 ± 1.8 (SD); p value = 0.012.	
Significant difference in correct answers between the treatment and the control groups for questions: 1) Does GDM increases the risk for the foetus to be macrosomic (67.5% versus 52.5%; p = 0.004) 5) Does GDM increases the risk for intrauterine foetal death (50% versus 30.5% p = 0.0001).	
Binomial logistic regression analysis: An affirmative answers to the questionnaire questions was more likely in the presence of a high level of education and the fact the fact of having had previous pregnancies.	

## Discussion

The aim of the present study was to evaluate the effectiveness of our post-diagnosis counselling in letting women diagnosed with GDM become aware of the foetal-maternal related risks.

Women who tested positive for GDM after receiving the post-diagnosis counselling have shown a secondary level of education, they gave up to 3 correct answers out of 5 at the questionnaire questions. In comparison with the control group, that did not receive any counselling, the treatment one knew more about the disorder. In particular, women in the treatment group demonstrated to be more aware about the risk to deliver a macrosomic foetus and to experience the most dramatic event for a pregnant woman: an intrauterine foetal death. Interestingly, risks such shoulder dystocia or polyhydramnios seemed not to have particularly captured women attention; an explanation can be postulated by the fact that the former is an infrequent event of whom women are not adequately aware in general, so it can be hard to visualize during the little time of counselling. Polyhydramnios, on the other hand, is a condition that can occur for many reasons, not only for GDM and this may have impacted on the decision making for the answer to the specific question. Preeclampsia is a well-known complication of pregnancy; an equal distribution in the aware women has been found in both groups. We decided to focus on foetal-maternal GDM related risks due to the evidence that women interest is captured by these topics, in particular foetal well-being. Indeed it has been previously demonstrated that “baby’s health” is the main motivational treatment factor for affected mothers [10, 11]. This data was confirmed in our study population, it has emerged that the arguments that most captured women attention during the post-diagnosis counselling have been those related to foetal risks. The treatment group was more aware than the control of the higher chance to deliver a foetus large for gestational age, moreover to experience the worst pregnancy outcome (intrauterine foetal death). We have also shown that the understanding of women with regards to specific foetal risks such as foetal macrosomia and intrauterine foetal death was positively affected by a high level of education and the fact of having had previous pregnancies. A high level of education may have allowed a better understanding of used terminology, and parity probably had given women the opportunity to get information about GDM during each of the previous pregnancies. The evidence of our research suggests that our post-diagnosis counselling has played an important role in improving women awareness about GDM related risks. This is a crucial result, since a stronger awareness of the possible repercussion on maternal and foetal well-being may improve women’s compliance to recommendations and definitively impact on glycaemic control and pregnancy outcome. Indeed, it is well known that a worse glycaemic control is associated with an almost 20% rate of preeclampsia, 25% rate of Caesarean section and increase of 2 to 4 fold the risk for foetal macrosomia and shoulder dystocia [1–3, 6]. Moreover, a worse glycaemic control is associated with a higher incidence of intrauterine foetal death. The latter not only impacts the mother and her foetus, but also the health care providers, both from a psychological prospective and a medico-legal one [15, 16]. A low level of awareness regarding GDM has been reported worldwide [10–14, 17], which shows us the importance to work on the best way to counsel women diagnosed with gestational diabetes mellitus. Our post-diagnosis counselling has had a positive impact on our treatment group. After receiving it, they’ve answered correctly to up to 3 questions out of 5 (secondary level of education), which is better than our control group. Despite the fact that we expected to obtain a tertiary level of education (5/5 correct answers), the medical terminology as well as the numerosity of concepts delivered all together may have played a role in the global understanding of women. Our study has shown how important is to make sure that women understand the information we give them, considering how fundamental their awareness about GDM related risks may be for pregnancy outcome. As a consequence of our study results, we’ve planned to ameliorate our counselling by implementing it with visual descriptions of concepts and explicative videos. The aim of each GDM clinic should be to sensitize women about the importance of this disorder as much as possible, especially about its related possible complications, considering the impact that awareness has on compliance to recommendations. Taking the high incidence of GDM across Italy into account, especially in southern regions [18], informative campaign involving consultants in universal validated counselling protocols should be realized.

The retrospective study design as well as the relatively low sample size limit the generalizability of the findings and may have led to potential biases and confounders; Moreover the difficulty in understanding of women about medical terminology and the emotional influence immediately after receiving the diagnosis of GDM, may have played a role in the final level of education.

## Conclusions

Awareness of foetal-maternal risks related to GDM is crucial to obtain the best compliance to recommendations among affected women. We realized a structured post-diagnosis counselling regarding GDM incidence, pathophysiology, risk factors, foetal and maternal risks and management that has shown to positively affect the level of maternal awareness regarding GDM related risks in particular those affecting foetal well-being.

Efforts should be employed to implement the post-diagnosis counselling with the aim to obtain a high level of awareness about this disorder and its related complications.

Further studies will be addressed to the realization and validation of an easier counselling, focusing on the feasibility for both the highly educated and the less educated population, furthermore to the realization of a study that will focus on the impact of pregnant women awareness on glycaemic control and pregnancy outcomes.

## Data Availability

The data that support the findings of this study are available from the electronic database of the Unit of Diabetology Pugliese Ciaccio Hospital, Catanzaro, Italy. Restrictions apply to the availability of these data, which were used under license for the current study, and so are not publicly available. Data are however available from the authors upon reasonable request and with permission of the Unit of Diabetology Pugliese Ciaccio Hospital, Catanzaro, Italy.

## Abbreviations

GDM: Gestational Diabetes Mellitus
OGTT: Oral Glucose Tolerance Test
T2DM: Type 2 Diabetes Mellitus
P.C: Patrizia Caroleo
P.Q: Paola Quaresima
L.P: Luigi Puccio
F.V: Federica Visconti
BMI: Body Mass Index
ORs: Odds Ratios
SD: Standard Deviation
